# WADD-SEPD Consensus on Psychological Treatment of Dual Disorders II: Neurodevelopmental, Anxiety, Post-Traumatic Stress, Somatic Symptom, Eating, and Personality Disorders and Recommendations for Future Research

**DOI:** 10.3390/jcm15031105

**Published:** 2026-01-30

**Authors:** Ana Benito, Susana Jiménez-Murcia, Judit Tirado-Muñoz, Ana Adan

**Affiliations:** 1 World Association on Dual Disorders (WADD), 28232 Madrid, Spain; judit.tirado@universidadeuropea.es (J.T.-M.); aadan@ub.edu (A.A.); 2 Spanish Society of Dual Disorders (SEPD), 28012 Madrid, Spain; sjimenez@bellvitgehospital.cat; 3 TXP Research Group, Medicine Department, Universidad Cardenal Herrera-CEU, CEU Universities, 12006 Castellón, Spain; 4 Torrente Mental Health Unit, General University Hospital of Valencia, 46900 Valencia, Spain; 5 Clinical Psychology Department, Bellvitge University Hospital-IDIBELL, 08907 Barcelona, Spain; 6 School of Medicine and Health Sciences, University of Barcelona, 08036 Barcelona, Spain; 7 Physiopathology of Obesity and Nutrition, CIBERobn, ISCIII, 28029 Barcelona, Spain; 8 Department of Psychology, Faculty of Biomedical and Health Sciences, Universidad Europea de Madrid, 28670 Madrid, Spain; 9 Network of Research in Primary Care of Addictions (RIAPAD), Carlos III Health Institute, 28029 Madrid, Spain; 10 Department of Clinical Psychology and Psychobiology, University of Barcelona, 08035 Barcelona, Spain; 11 Institute of Neurosciences, University of Barcelona, 08035 Barcelona, Spain

**Keywords:** dual disorders, dual diagnosis, neurodevelopmental disorders, anxiety disorders, post-traumatic stress disorder, somatic symptom disorders, eating disorders, personality disorders, psychological treatment, psychotherapy

## Abstract

**Background/Objectives**: The treatment of dual disorders (DDs) must be comprehensive and multidisciplinary. Evidence supports the effectiveness of psychotherapy in treating DDs. The second part of this consensus synthesizes the available evidence on psychological treatment for specific DDs. **Methods**: Two consensus methods were sequentially implemented: the nominal group technique and the Delphi method. **Results**: This consensus review encompassed a compilation of recommendations for the psychological treatment of neurodevelopmental, anxiety, post-traumatic stress, somatic symptom, eating, and personality disorders. Finally, recommendations for the future research agenda on the psychological treatment of DD were included. **Conclusions**: (1) Psychological treatment, particularly integrated treatment, is effective. (2) In the case of dual autism, interventions for substance use disorders should be adapted to this population’s characteristics. (3) More research is needed on dual social anxiety, panic, generalized anxiety, somatic symptom, and eating disorders, for which Cognitive Behavioral Therapy (CBT) is the most commonly used treatment. (4) For dual attention deficit hyperactivity disorder, multicomponent treatment is recommended (psychoeducation, CBT, and peer or family support). (5) For dual anxiety disorders, CBT is the first-line treatment. (6) For dual post-traumatic stress disorder, CBT (cognitive processing therapy and prolonged exposure therapy), acceptance and commitment therapy, stress inoculation training, and Eye Movement Desensitization and Reprocessing (EMDR) are effective. (7) For dual personality disorders, evidence is scarce. (8) For borderline personality disorder, dialectical behavior therapy, dynamic deconstructive psychotherapy, and dual-focus schema therapy show promise. (9) For antisocial personality disorder, CBT, contingency management, and counseling on impulsive lifestyles may be useful. (10) Much more evidence is needed from studies that overcome the methodological limitations of existing ones.

## 1. Introduction

Dual disorders (DDs) refer to the presence of both addiction and another mental disorder (MD), either simultaneously or sequentially throughout an individual’s lifetime [1]. DDs are highly prevalent and have significant repercussions [2,3,4]. Estimates suggest that over 90% of adults with MDs and substance use disorders (SUDs) do not receive adequate treatment [5]. DDs treatment should be comprehensive and involve multidisciplinary teams [6]. Although psychotherapy has been proven effective in this context [7], clinical guidelines, consensus statements, and reviews on DDs treatment [8], based on a combination of expert opinions, clinical experience, and research evidence [9], tend to dedicate considerably less attention to psychological therapy than to pharmacological therapy.

In the psychological treatment of DD, the controversies and research gaps that exist in pharmacological treatment are replicated: parallel versus integrated treatment [7]; exclusion of subjects with DD from studies; lack of randomized clinical trials; heterogeneity of studies (different samples, SUDs, MDs, outcomes, etc.); lack of definition of what constitutes standard treatment; low methodological quality of studies; and understudied populations, comorbidities, and barriers to treatment [5,9]. Furthermore, studies on psychological treatment present their own controversies and gaps: what is standard psychological treatment; what components each therapy has; which therapy is most effective for which disorders; studies with smaller samples and less methodological rigor; and high experimental mortality [4,5,9].

Therefore, the second part of this consensus synthesizes the available evidence on specific DDs, including neurodevelopmental disorders, anxiety disorders, post-traumatic stress disorder (PTSD), somatic symptoms, eating disorders, and personality disorders (PDs), and provides general recommendations for a future research agenda that advances knowledge of the clinical experience of psychological treatment for DDs.

## 2. Materials and Methods

Two consensus methods were implemented sequentially: the nominal group technique and the Delphi method [10]. The methodology was described in detail in the first part of this work [11]. All data and evidence included had to have the unanimous consensus of the authors. The document was reviewed in successive versions until the final content was approved. If there had been any disagreement, that content would not have been included in the consensus, although this did not happen. The second part of the consensus includes recommendations for the psychological treatment of specific DDs (applicable to the MDs in the DSM-5-TR [12], listed in the order they appear, and, within each disorder, recommendations applicable to all SUDs, followed by those applicable to each specific SUD, listed in the order they appear in the DSM-5-TR) [12]. Thus, neurodevelopmental disorders, anxiety disorders, PTSD, somatic symptoms, eating disorders, and PDs have been included. Finally, the last section presents general recommendations for future research. For each of the MDs considered, if the heading referring to its comorbidity with a specific SUD does not appear, it means that no studies on the subject have been identified. Likewise, if an MD does not appear, no recommendations are available for it. When referring to standard treatment, it generally refers to core components of single-disorder therapies based on existing guidelines. When referring to integrated treatment, it means treatment provided by the same team of professionals who manage both disorders within the same setting [3]. Concurrent treatment (treating both disorders at the same time) is often used as a synonym for integrated treatment, although it can sometimes be confused with parallel treatment (each disorder is treated by a different team at the same time).

The level of evidence rating (when available) is provided in parentheses. These ratings are interpreted as follows [13]: 1—well-designed randomized, controlled clinical trials or meta-analyses; 2—clinical trials with minor methodological limitations; 3—descriptive, comparative, and case–control studies; 4—consensus of expert committees, technical reports from health authorities, and case series. The strength of the available recommendations, also indicated in parentheses, is interpreted as follows: A—Maximum; B—High; C—Medium; D—Low [13,14]; Strong; Weak [15]. When no level or grade is specified, it should be considered as level 4, grade D, and a weak recommendation.

## 3. Results

*Recommendations for the Psychological Treatment of Specific Dual Disorders*

In the absence of research on specific comorbid disorders, the most effective treatments for each disorder are recommended [9].

### 3.1. Neurodevelopmental Disorders

#### 3.1.1. Autism Spectrum Disorders

- Substance-related disorders

For children, young people, and adults with autism spectrum disorders, the National Institute for Health and Clinical Excellence (NICE) guidelines [16] recommend offering interventions for the specific disorder, in this case, SUD, adapted to the characteristics of this population, as is done for other comorbid MDs [17].

#### 3.1.2. Attention Deficit Hyperactivity Disorder

A summary of evidence and recommendations is provided in Table 1.

- Substance-related disorders

There is insufficient evidence regarding the psychological treatment of dual attention deficit hyperactivity disorder (ADHD) [15]. Few studies have explored the treatment of patients with comorbid ADHD and SUD [5], and studies evaluating non-pharmacological interventions are scarce [18,19].

A combination of psychoeducation (PE), behavioral coaching, cognitive behavioral therapy (CBT), and non-stimulant or long-acting stimulant medication is recommended [5]. Psychotherapeutic interventions, such as PE, CBT, motivational therapy, and relapse prevention (RP), are considered essential elements in the comprehensive management of both adolescent and adult patients with dual ADHD [19]. In addition, traditional psychological interventions for SUDs are considered effective treatments for these DDs [20], and skills training based on dialectical behavior therapy (DBT) [21] is a promising treatment [9].

The NICE [22] guideline identifies CBT as the preferred non-pharmacological treatment. Expert consensus recommends a multimodal treatment approach combining medication and CBT; however, success depends heavily on the content of the cognitive behavioral intervention [18]. CBT is the non-pharmacological treatment supported by the most evidence for treating dual ADHD in adolescents and adults. Combining CBT with pharmacological treatment significantly improves ADHD and SUD symptoms, regardless of whether patients receive effective pharmacological treatment or placebo [19]. There is also preliminary evidence supporting the use of behavioral interventions focused on academic training for adolescents with ADHD and SUD; however, further research is needed [9].

Family involvement in the treatment process is also recommended, as peer and family support can enhance its effectiveness (level of evidence: D) [14]. Another recommendation is adapting interventions to the executive functioning deficits associated with ADHD, such as difficulties with attention, impulse control, organization, and time management. These adaptations include shorter and/or more frequent sessions, reminders to reduce absences due to forgetfulness, setting specific deadlines, greater use of supplementary visual materials, highly structured therapy, and using a notebook during sessions [20].

An integrated treatment model for patients with ADHD and SUDs has been proposed: integrated CBT (iCBT) [23]. However, its results have been modest [19]. Although a greater reduction in ADHD symptoms was observed in participants in the integrated group, no differences were found in substance use or other outcomes [9].

In this regard, there is little evidence supporting the simultaneous treatment of ADHD and SUDs [5], and there is no consensus on the order in which to treat comorbid disorders [14]. Some researchers recommend addressing the more debilitating disorder first. Others suggest prioritizing the treatment of SUDs to stabilize the patient, given the potential for harmful interactions between ADHD medication and the substance used [5], followed by ADHD treatment as soon as possible [18]. In contrast, some authors recommend an integrated, multimodal approach, with components of individual and/or group psychotherapy (including goal-focused CBT), PE, and peer and family support [9]. In this regard, it is important to remember that effective ADHD treatment does not usually improve SUDs, so it is crucial to treat both disorders effectively [18]. While more research is needed, a comprehensive approach to treating substance use and ADHD is recommended (level of evidence: C) [14].

According to Matthys et al. [14], abstinence is not a prerequisite for initiating treatment; however, substance use must be stabilized (level of evidence: D). Furthermore, when SUD symptoms are too severe or housing is unstable, short-term hospital or residential treatment is recommended.

In summary, there is a general consensus that the appropriate ADHD treatment for individuals with SUD includes several components: PE and medication should be complemented by individual or group CBT, as well as peer or family support [14,18]. Research suggests that combined pharmacotherapy and psychotherapy are useful in adults [18], achieving better results than medication alone (level of evidence: D) [14]. Therefore, according to the guidelines by Matthys et al. [14], multimodal treatment is recommended, with the implementation of as many interventions as possible: incorporating PE in the first phase and CBT and skills training (individual or group) in the second phase (level of evidence: C). DBT [21] and mindfulness (MF) training may also be useful, and couples therapy should be considered (level of evidence: D).

- Alcohol-related disorders

Treatment in adolescence and early adulthood should be patient-oriented and multidimensional, considering psychosocial, psychoeducational, and family factors as part of a comprehensive approach [24].

**Table 1:** Recommendations for the psychological treatment of dual attention deficit hyperactivity disorder.

Attention Deficit Hyperactivity Disorder		
Substance-Related Disorders	Recommended Therapies	Specific Recommendations
**Substance-related disorders**	Limited evidence. CBT has the strongest evidence and is preferred. Combine as many interventions as possible: psychoeducation, behavioral coaching, individual and/or group CBT, motivational interviewing, relapse prevention, skills training, peer support, and family support. Dialectical behavior therapy, mindfulness, and couples therapy may be helpful.	Adults: A combination of pharmacotherapy and psychotherapy yields better results than medication alone. Adolescents: Psychoeducation, CBT, motivational interviewing, relapse prevention, and behavioral interventions focused on academic development.
**Alcohol-related disorders**	Comprehensive multimodal therapeutic approach.	Adolescents and young adults: patient-oriented and multidimensional treatment.

Note: Prepared by the authors based on [5,9,14,18,19,20,22,23,24]. CBT: cognitive behavioral therapy.

### 3.2. Anxiety Disorders

Table 2 provides a summary of evidence and recommendations.

- Substance-related disorders

Few studies have examined the psychological treatment of anxiety disorders with comorbid SUDs, and the available data are inconclusive or contradictory [9,25,26]. Evidence suggests that the standard treatment for SUDs is effective for individuals with co-occurring anxiety [27]. In fact, most published data are based on CBT [28,29]. Integrated treatments that include CBT or exposure therapy (ET) can safely and effectively reduce psychiatric and SUD symptoms. However, in some studies, these treatments have shown no greater effectiveness than placebo [5].

Recommended integrated treatments include educational interventions on the nature of anxiety, CBT (anxiety monitoring, thought restructuring, clarification of cognitive distortions, emotional intelligence, and relaxation training), motivational support, mental health support, and promotion of a healthy lifestyle, along with medication when necessary [5].

In most cases, CBT is the first-line treatment, as it is more cost-effective and efficient than medication at reducing anxiety symptoms, particularly for individuals with little or no prior exposure to benzodiazepines. Furthermore, CBT is more effective when sedation and anxiolysis due to benzodiazepine use are minimal [30].

In summary, there is growing evidence suggesting that integrated treatment approaches can effectively reduce symptoms of anxiety, depression, stress, and substance use while improving quality of life for individuals with dual anxiety [9].

- Alcohol-related disorders

CBT combined with motivational interviewing (MI) [31] has shown efficacy, especially in long-term intervention programs associated with greater long-lasting effectiveness [26,32]. However, some studies have found no advantage to concurrent psychological treatment [25].

CBT is recommended to reduce alcohol consumption and the level of internalization (anxiety and depression) (weak recommendation) [15], although successful treatment of anxiety or alcohol use disorder with CBT does not necessarily result in a positive outcome for the comorbid disorder [30]. For patients with these DDs, CBT is more effective in improving SUDs in individuals who drink less and should be considered before benzodiazepine treatment to reduce anxiety [30]. A meta-analysis by Preuss et al. [24] obtained Cohen’s d effect sizes of 0.66 for CBT and 0.24 for medication.

- Cannabis-related disorders

CBT is the most commonly used treatment for cannabis use and is effective in reducing anxiety symptoms [30].

- Inhalant-related disorders

Gordon’s [30] recommendations, which are heavily based on the type of substance used, with less emphasis on comorbidity, consist of using standard CBT approaches, such as assertiveness and coping skills or alternatives to substance use, and complementing them with community reinforcement, family interventions, and an assertive approach.

- Opioid-related disorders

Studies are needed to determine the effectiveness of interventions for people with opioid use disorder and anxiety disorders [33].

- Disorders related to sedatives, hypnotics, or anxiolytics

There is no evidence for any specific treatment for this comorbidity [34]. CBT and gradual exposure may be effective when administered alongside benzodiazepine dose reductions. This combination has been shown to increase the likelihood that patients with an anxiety disorder will successfully reduce and discontinue benzodiazepine use [30].

- Stimulant-related disorders

CBT is effective in reducing general anxiety symptoms [30]; there is no recent evidence allowing us to go beyond this general statement.

#### 3.2.1. Social Anxiety Disorder (Social Phobia)

- Alcohol-related disorders

There is insufficient evidence to support a specific recommendation [35]. Marel et al. [9] reviewed three randomized controlled trials examining the efficacy of CBT-based therapies, which yielded mixed results. The authors suggest that integrated or complementary treatment of social anxiety and alcohol use disorder may be more beneficial than treating alcohol use alone. However, further research is needed regarding the impact of incorporating CBT. They also emphasize the importance of exploring the potential efficacy of other types of psychotherapy, as existing studies have focused on CBT due to the strong evidence supporting its effectiveness in treating social anxiety as a standalone disorder.

- Cannabis-related disorders

CBT focused on coping mechanisms will be more effective for people who have used cannabis or other hallucinogens to self-medicate and cope with situations of social anxiety [30].

- Stimulant-related disorders

Similar to the treatment of cannabis-related disorders, CBT focused on coping mechanisms will be more effective if stimulants are used as a form of self-medication or coping in situations of social anxiety [30].

#### 3.2.2. Panic Disorder

- Substance-related disorders

Few studies have examined the treatment of panic disorder when it coexists with SUDs. These studies have focused on CBT and produced preliminary findings. In the absence of sufficient evidence, strategies similar to those proven effective in treating panic disorder alone are appropriate. These strategies include a stepped-care approach beginning with ET and lifestyle advice, followed by CBT, pharmacotherapy supplemented with gradual exposure, or a combination of CBT and pharmacotherapy as needed, depending on symptom severity [9].

- Alcohol-related disorders

There is no evidence to support a specific recommendation [35].

#### 3.2.3. Generalized Anxiety Disorder

- Substance-related disorders

There is a lack of evidence on the effectiveness of psychological therapies for this comorbidity [9].

- Alcohol-related disorders

There is no evidence to support a specific recommendation [35].

- Opioid-related disorders

These patients can benefit from relaxation techniques, training in coping skills, cognitive restructuring, behavioral activation, problem solving, and improved sleep hygiene [33].

**Table 2:** Recommendations for the psychological treatment of dual anxiety disorders.

Anxiety Disorders		
Substance Related Disorders	Recommended Therapies	Specific Recommendations
**Substance-related disorders**	The standard treatment for SUDs is effective. The strongest evidence supports CBT. Psychoeducation, CBT, exposure therapy, motivational training, mindfulness, and promoting a healthy lifestyle, along with medication when necessary, are effective.	
**Alcohol-related disorders**	CBT combined with motivational interviewing is effective. CBT is recommended as the first line of treatment before considering benzodiazepines.	CBT is more effective for people who drink less.
**Cannabis-related disorders**	Effective CBT.	
**Inhalant-related disorders**	CBT combined with community reinforcement, family interventions, and an assertive approach.	
**Opioid-related disorders**	Studies are needed.	
**Disorders related to sedatives, hypnotics or anxiolytics**	CBT and exposure therapy are effective when administered concurrently with benzodiazepine dose reductions.	
**Stimulant-related disorders**	Effective CBT to reduce general anxiety symptoms.	
**Social Anxiety Disorder (Social Phobia)**		
**Substance-Related Disorders**	**Recommended Therapies**	**Specific Recommendations**
**Substance-related disorders**	CBT is the most commonly used treatment.	
**Panic Disorder**		
**Substance-Related Disorders**	**Recommended Therapies**	**Specific Recommendations**
**Substance-related disorders**	CBT is the most used. Stepwise approach: psychoeducation, cognitive behavioral therapy, and pharmacotherapy complemented with gradual exposure.	Depending on the severity of the symptoms, a combination of CBT and pharmacotherapy.
**Generalized Anxiety Disorder**		
**Substance-Related Disorders**	**Recommended Therapies**	**Specific Recommendations**
**Substance-related disorders**	Evidence is lacking.	
**Opioid-related disorders**	Relaxation techniques, coping skills training, cognitive restructuring, behavioral activation, problem solving, and sleep hygiene.	

Note: Prepared by the authors based on [5,9,15,26,27,28,29,30,32,33,34,35]. CBT: cognitive behavioral therapy. SUD: substance use disorders.

### 3.3. Post-Traumatic Stress Disorder

A summary of evidence and recommendations is provided in Table 3.

- Substance-related disorders

Various psychotherapeutic interventions have been developed to treat PTSD comorbid with SUDs, and interest in this area has increased in recent years [9]. While there is some controversy regarding nomenclature conventions, the existing approaches can be divided into two types of therapy: past/trauma-focused and present/non-trauma-focused [9]. Past/trauma-focused therapy involves reviewing traumatic memories and their meaning. In contrast, present/non-trauma-focused therapy focuses on developing coping skills in the present, without an in-depth discussion of the trauma [36].

Evidence-based trauma-focused psychotherapies for PTSD, such as Cognitive Processing Therapy (CPT) [37] and Prolonged Exposure (PEx) [38], demonstrate comparable treatment completion and tolerability rates across various SUD subtypes. However, CPT is associated with a higher likelihood of treatment completion compared to PEx [39].

CPT and CPT integrated with CBT for substance use have shown promising results in individuals with SUD; however, research has primarily been conducted in veterans [9]. Studies comparing CPT outcomes in veterans with and without comorbid SUDs have found no significant differences between the groups. Subsequent open-label studies of CPT combined with CBT for substance use have also reported reductions in PTSD symptoms, depressive symptoms, and substance use-related outcomes [9].

The PEx [38] was modified to include sessions addressing substance use with CBT strategies, resulting in the Concurrent Treatment of PTSD and SUDs Using PEx (COPE) [40]. This approach can be augmented with cognitive behavioral treatment for SUDs [36,41]. COPE appears to result in greater improvements in PTSD and SUDs than usual treatment [33]. Studies have shown that although the reduction in substance use is comparable to that of controls, the program achieves greater reductions in PTSD symptoms compared to usual treatments for substance use, RP, and present-focused/non-trauma Seeking Safety therapy [9,42], which will be discussed later.

Preliminary evidence suggests that CPT and PEx can be administered to patients with dual PTSD, showing good acceptance and improvements in both PTSD and SUDs [43]. Although more evidence is needed, PEx may be safe and effective in reducing trauma and SUD symptoms [5]. Additionally, there are positive, albeit preliminary, data indicating that Eye Movement Desensitization and Reprocessing (EMDR) [44], when combined with schema therapy for PTSD and SUDs, may effectively reduce both PTSD and substance use [9].

Two other exposure-based behavioral therapies developed specifically for dual PTSD have been studied: PTSD with substance dependence treatment [45] and concurrent treatment of PTSD and cocaine dependence [46,47]. Both have been investigated in small, uncontrolled clinical trials [43].

Due to concerns that exposure to trauma cues could trigger relapse, Seeking Safety [42] has been used. This is a manualized cognitive behavioral intervention for women with comorbid PTSD and SUDs. This intervention does not involve exposure to trauma-related cues or narratives [33]. Several uncontrolled pilot studies initially suggested that Seeking Safety could effectively reduce substance use and, in some cases, PTSD symptoms [43]. Subsequent randomized controlled trials and meta-analyses show that treatment outcomes for PTSD and substance use are better than those of patients who receive no treatment but are comparable to outcomes of patients who receive other treatments, such as RP, typical substance use treatment, or health education [9].

An emerging therapy focused on the present is integrated CBT (ICBT) [48]. Two randomized clinical trials have examined its effectiveness compared to usual care and individualized addiction counseling. These trials found no significant differences in PTSD outcomes, but one observed improvements in substance use [9].

Non-exposure treatments may be moderately effective in alleviating symptoms of both PTSD and substance use, but the evidence is still preliminary [5]. Approaches typically used for alcohol and other drug problems are also being investigated in individuals with this comorbidity. Studies using CBT have shown positive and encouraging efficacy results [28,43]. Based on the current (albeit limited) evidence, it is recommended that clinicians adapt RP methods to help patients with SUDs identify trauma-related relapse signals and prepare them to cope with those triggers without using substances [43].

MF-based programs have also shown promise in early pilot studies. One randomized clinical trial found that participants in the MF-Oriented Recovery Enhancement program [49] showed greater improvements in PTSD symptoms, alcohol cravings, and negative affect compared to participants in the Seeking Safety program [9]. A small-sample study of veterans using Mindful Self-Compassion revealed a decrease in PTSD symptoms and the number of drinking days [50].

Integrated and concurrent treatment, which addresses both MDs simultaneously, has received strong empirical support. It is also preferred by patients and is increasingly considered the current standard of care, especially when combined with psychosocial and pharmacological approaches [5]. Examples of concurrent treatments include COPE [40], ICBT [51], and Seeking Safety [52]. Another example is trauma-integrated family therapy-based RP for women with PTSD and SUD (TI-MBRP) [53], which showed significant reductions in PTSD severity and craving over a twelve-month period [53]. Although this approach was acceptable to participants, it had a high dropout rate (64%) at the one-year follow-up.

In a sample of individuals with PTSD seeking treatment for SUD, trauma-focused motivational enhancement therapy was associated with significantly greater reductions in PTSD symptoms and positive urine drug tests compared to a control condition [5]. RP therapy combined with ET was associated with marked improvements in SUD and PTSD symptom severity, as well as reduced substance use in the previous week [5].

Another promising integrated exposure-based program for adolescents is Risk Reduction through Family Therapy [54], which combines trauma-focused CBT and multisystemic therapy. This program has shown promise in reducing PTSD symptoms, substance use, and risk behaviors [9].

It is important to highlight that interventions that address both PTSD and SUD concurrently are effective and safe, with no observed increase in substance use or worsening of PTSD [55]. Despite the effectiveness of concurrent treatment, individuals with PTSD and SUDs often only receive addiction treatment. Furthermore, it is common that patients in SUD treatment settings are not assessed for a possible PTSD diagnosis [5]. While SUD treatment alone rarely improves PTSD symptoms, addressing these symptoms significantly decreases the likelihood of excessive substance use [5].

Several reviews have concluded that past/trauma-focused individual psychological interventions using exposure-based approaches are supported, particularly in relation to PTSD outcomes. In contrast, there is very little evidence to support the use of non-trauma-focused individual or group interventions instead of standard treatments for substance use [9].

Some authors recommend interventions that integrate exposure-based PTSD treatment with behavioral therapy for SUD, as they are associated with better PTSD outcomes than SUD treatment matched for time and attention [36]. These integrated programs usually include PE for each disorder and its interrelationship, coping skills training, RP, and exposure to traumatic memories or reminders. They are sometimes implemented in combination with other therapeutic techniques [9]. Support for these programs is growing, with an increasing number of studies providing evidence of their efficacy and safety. Improvements in substance use and PTSD symptoms have been observed, and no high relapse rates or worsening of symptoms have been observed during follow-up [9]. However, other authors conclude that interventions with integrated versus non-integrated psychological treatments cannot be recommended for dual PTSD (weak recommendation) given that there is similar improvement in the symptomatology of both disorders [15].

The authors agree that these patients can benefit from various therapeutic options, including standard treatment for SUDs [9,36]. Various psychological treatments, such as CBT and Acceptance and Commitment Therapy (ACT) [56], are effective in treating patients with SUDs and PTSD (level 3 evidence) [13,43]. Preliminary results suggest that evidence-based psychotherapies recommended for PTSD, such as CPT, PEx, stress inoculation training, and EMDR, may be effective in treating PTSD comorbid with SUDs. However, limited data make it difficult to clearly identify a specific treatment as the “gold standard” [43]. It is important to emphasize that, although there is a solid evidence base for certain treatments, treatment plans must be individualized to each patient [9].

- Alcohol-related disorders

As Taylor et al. [57] pointed out in their review, ET (imaginal or prolonged), CPT, and CBT have been evaluated and found to be effective and safe. There is no indication that alcohol consumption increases with these interventions. However, while these interventions are effective in reducing alcohol consumption and PTSD symptoms, the outcomes are not superior to those observed in a control group. This may be due to study limitations, as most have had inadequate sample sizes and control groups.

Trauma-focused therapy reduces both PTSD symptoms and alcohol consumption in the long term, although the evidence is weak [35].

Although research examining the effectiveness of ACT is in its early stages, it has been associated with improvements in PTSD symptoms and reductions in both the frequency and amount of alcohol consumption in veterans with dual PTSD [9].

A controlled study on the treatment of PTSD comorbid with alcohol use disorder showed efficacy only for CBT [24]. Patients in the intervention group who received at least one ET session showed more than twofold improvement in PTSD symptoms and reduced alcohol consumption five months after the treatment ended compared to the control group. Another randomized study of an integrated stabilizing CBT program (without exposure) showed improvements in both PTSD symptoms and substance use at the 12-month follow-up [24].

**Table 3:** Recommendations for the psychological treatment of dual post-traumatic stress disorder.

Post-Traumatic Stress Disorder		
Substance-Related Disorders	Recommended Therapies	Specific Recommendations
**Substance-related disorders**	Cognitive processing therapy and prolonged exposure therapy are effective, acceptable and safe. Integrated and concurrent treatment is effective and safe. COPE shows greater improvements in both disorders than usual care. Preliminary efficacy data is available for EMDR combined with schema therapy. Treatments without exposure may be moderately effective. Mindfulness-based programs, trauma-focused motivational enhancement therapy, and relapse prevention with prolonged exposure show promise. Effective cognitive processing therapy, prolonged exposure, CBT, acceptance and commitment therapy, stress inoculation training, and EMDR.	For veterans, cognitive processing therapy results in no difference whether comorbid substance use disorder is present or absent. For women, Seeking Safety is more effective than no treatment but comparable to relapse prevention, standard treatment for substance use, or health education. The Risk Reduction through Family Therapy program shows promise in adolescents.
**Alcohol-related disorders**	Exposure therapy (imaginal or prolonged), cognitive processing therapy, and cognitive behavioral therapy are safe and effective. Trauma-focused therapy shows weak evidence of effectiveness. CBT, with or without exposure, shows stronger evidence of efficacy.	In veterans, acceptance and commitment therapy is effective.

Note: Prepared by the authors based on [5,9,13,24,28,33,35,39,40,42,43,53,55,57]. CBT: cognitive behavioral therapy. COPE: Concurrent Treatment of Post-Traumatic Stress Disorder and Substance Use Disorders Using Prolonged Exposure [40]; EMDR: Eye Movement Desensitization and Reprocessing [44].

### 3.4. Somatic Symptom Disorders and Related Disorders

- Inhalant-related disorders

The lack of studies on the treatment of these DDs was first highlighted almost twenty years ago [30] and persists to this day.

- Opioid-related disorders

The non-pharmacological management of somatoform disorders should be emphasized as the first-line treatment, as CBT has been shown to reduce somatic symptoms and improve functioning. This recommendation is supported by the requirement that a clinical psychologist experienced in pain management be involved [30].

- Disorders related to sedatives, hypnotics, or anxiolytics

Provided that resources are available and the patient is willing to participate, it is essential to prioritize non-pharmacological management of the somatoform disorder as the first-line treatment [30].

CBT has demonstrated a reduction in somatic symptoms and an improvement in functioning in individuals with dual somatoform disorders. Reducing the dosage of benzodiazepines or other sedative hypnotics is necessary for CBT to be more effective [30].

- Stimulant-related disorders

According to Gordon [30], brief interventions and MI are recommended for stimulant use. He indicates that CBT can address stimulant use and somatoform disorder, though no studies have demonstrated its effectiveness in managing these DDs.

### 3.5. Eating Disorders

Table 4 provides a summary of evidence and recommendations.

- Substance-related disorders

There is very limited evidence, and no randomized controlled trials have been conducted, regarding the treatment of this comorbidity [5,9]. Integrated treatment is generally recommended, as sequential interventions may increase the likelihood of relapse or hinder recovery from the untreated disorder [5]. If integrated care is not possible, the Substance Abuse and Mental Health Services Administration [5] recommends initiating treatment for the SUD first to stop active substance use and enable the patient to fully participate in subsequent care. Regardless of the treatment modality, ensuring medical and weight stabilization first is crucial so that patients can physically and cognitively participate in and benefit from therapy. Some patients with anorexia or bulimia nervosa will require prior inpatient treatment or partial hospitalization to stabilize their weight.

The primary treatment for these disorders is psychosocial intervention, including individual, group and family therapies, or a combination of these therapies [5]. Preliminary evidence suggests that structured programs incorporating elements of established psychotherapies, such as CBT, family therapy, and DBT [21], can effectively reduce eating disorder symptoms in individuals with SUD [9]. There is also evidence suggesting that treating SUD can improve eating disorder symptoms [9].

CBT may be effective for eating disorders, but its effectiveness in populations with a comorbid SUD has not been thoroughly investigated [5]. DBT [21] may also be useful in eating disorders and SUDs separately, but again, its effectiveness in patients with DDs has not been thoroughly studied [5].

Several specific therapeutic approaches have been designed for this comorbidity, such as a CBT approach based on MF action [58], a DBT approach [59], and a group therapy model that uses a gender-specific CBT approach [60]. These integrated approaches are associated with reductions in binge eating, cravings, and addiction severity [61]. Thus, they appear to be useful and require further study.

In summary, there is little evidence to provide clear guidance on the treatment of this comorbidity. Research on standalone eating disorders suggests that comprehensive treatment by a multidisciplinary team should include psychotherapy as the first line of treatment, with CBT-based approaches having the strongest evidence [9].

- Alcohol-related disorders

CBT is effective for treating eating disorders, particularly bulimia. There is no evidence that alcohol dependence negatively affects its effectiveness [30].

Support for stress management has been shown to be effective in treating alcohol use disorder and is helpful for individuals with eating disorders [30]. This may be applicable to patients with DDs, although further studies are needed to demonstrate its effectiveness, taking into account gender and using the longest possible follow-up period, given the characteristics of both disorders.

- Cannabis-related disorders

In the absence of other proven treatments, CBT is currently the most widely used psychotherapeutic treatment modality for cannabis use [30].

- Inhalant-related disorders

As in other DDs involving inhalant-related disorders, standard CBT approaches should be used. These approaches include assertiveness and coping skills training, as well as alternatives to substance use, supplemented with community reinforcement, family interventions, and an assertive approach [30].

- Stimulant-related disorders

CBT can be used to address stimulant use and eating disorders. It appears to be helpful in providing coping strategies for impulsive stimulant use and binge eating [30].

#### 3.5.1. Anorexia

- Substance-related disorders

There are no evidence-based psychotherapies for this comorbidity [9].

#### 3.5.2. Bulimia

- Substance-related disorders

CBT has proven useful in treating bulimia and is generally considered the first-line treatment [30]. There is preliminary evidence suggesting that combining CBT with medication is more effective, although further studies are needed to confirm this [30]. There is limited evidence for the simultaneous treatment of SUDs and bulimia nervosa, though it has been suggested that treating SUDs may improve bulimia [9].

#### 3.5.3. Binge Eating Disorder

- Substance related disorders

Currently, there is no evidence-based psychotherapy for treating this comorbidity [9].

**Table 4:** Recommendations for the psychological treatment of dual eating disorders.

Eating Disorders		
Substance-Related Disorders	Recommended Therapies	Specific Recommendations
**Substance-related disorders**	Integrated treatment is recommended, but if it is not possible, SUD should be treated first. The priority is medical and weight stabilization. The main treatment is psychosocial intervention: individual, group, family therapy, or a combination. Structured programs (CBT, family therapy, and dialectical behavior therapy) can effectively reduce eating disorder symptoms. Integrated therapeutic approaches, mindfulness-action-based CBT, integrated dialectical behavior therapy, and group therapy with a gender-specific CBT approach decrease binge eating, cravings, and addiction severity.	Some patients may require hospital treatment or partial hospitalization to stabilize their weight.
**Alcohol-related disorders**	CBT is effective for eating disorders, particularly bulimia, with no negative effects if alcohol dependence is present. Effective stress management support for treating alcohol abuse and helpful for people with eating disorders.	
**Cannabis-related disorders**	CBT is the most commonly used.	
**Inhalant-related disorders**	Standard approaches to cognitive behavioral therapy, such as assertiveness and coping skills training and alternatives to substance use, may be complemented by community reinforcement, family interventions, and an assertive approach.	
**Stimulant-related disorders**	CBT may be helpful. Assistance with coping strategies may help with impulsive stimulant use and binge eating.	
**Anorexia**		
**Substance-Related Disorders**	**Recommended Therapies**	**Specific Recommendations**
**Substance-related disorders**	There are no evidence-based psychotherapies.	
**Bulimia**		
**Substance-Related Disorders**	**Recommended Therapies**	**Specific Recommendations**
**Substance-related disorders**	Combining CBT and medication is more effective. Treating SUD may improve bulimia.	
**Binge Eating Disorder**		
**Substance-Related Disorders**	**Recommended Therapies**	**Specific Recommendations**
**Substance-related disorders**	There are no evidence-based psychotherapies.	

Note: Prepared by the authors based on [5,9,30,58,59,60,61]. CBT: cognitive behavioral therapy. SUD: substance use disorders.

### 3.6. Personality Disorders

Table 5 provides a summary of evidence and recommendations.

- Substance-related disorders

Although the interventions differ from those used for other DDs, individuals with PD and SUD can be treated effectively. As Becoña and Cortés [62] point out, the following factors should be considered: (1) PD progression establishes a specific rhythm to which therapeutic strategies must be adapted; (2) individuals with PD may present different substance use patterns that could affect rehabilitation programs; (3) interventions should be guided by personality; and (4) long-term treatment with good adherence is required. CBT is effective in treating SUDs when the patient has a PD, although to evaluate its effectiveness, variables related to addiction, personality traits, and quality of life must be taken into account.

Other particularities of the psychological treatment of individuals with dual PDs [62,63] include the following: the recommended time between sessions should be as short as possible; the duration of the sessions depends on the PD; family members need a higher level of education; the relationship between the patient and therapist must take into account different aspects depending on the type of PD; a greater degree of dedication and specialization is required of the professional; the impact of sporadic substance use on the evolution of the treatment increases the probability of relapse; treatments should be longer even if abstinence has been achieved; follow-up sessions after discharge are very important, especially in borderline personality disorder (BPD) or dependent PD; and RP programs must be adapted to the characteristics of these patients.

The therapeutic alliance is the most significant predictor of the intervention outcome and the quality of the therapeutic relationship, which will improve if it is guided by the patient’s personality [64].

Although there are no specific, scientifically based treatments for PDs, there are effective treatments for addressing various symptoms, such as the risk of suicide and self-harm, affective dysregulation, maladaptive thought patterns, and interpersonal dysfunction [5]. Psychotherapy is the primary form of intervention since no medications have been approved for the treatment of PDs.

DBT [65], dynamic deconstructive psychotherapy [66], and dual-focus schema therapy [67] appear promising, especially for dual BPD. These therapies have demonstrated a positive effect on psychiatric and addiction-related outcomes [68]. However, overall, research on effective treatments for PDs, with or without co-occurring SUDs, is limited and requires further evidence [5].

Experts suggest that treating PDs and SUDs simultaneously, using a combination of psychotherapy and pharmacotherapy to support the reduction or cessation of substance use, may be the best approach, although research comparing this approach with others is lacking [9].

A staged approach has also been recommended. The initial phases of treatment focus on stabilizing substance use and self-harm behaviors using a transdiagnostic approach centered on emotion regulation and impulsivity, followed by interventions focused on issues related to identity and self [9]. Schema-focused therapy is particularly useful for treating dual PDs that do not respond to brief intervention [68].

- Alcohol-related disorders

No evidence-based intervention was found [69].

- Disorders related to sedatives, hypnotics, or anxiolytics

There is no evidence of a specific treatment for this comorbidity [34].

- Cannabis-related disorders

In the absence of other proven, effective treatment methods, CBT is currently the most widely used treatment for cannabis use [30].

- Stimulant-related disorders

CBT is effective in reducing stimulant use. In particular, assistance with coping strategies has proven useful in cases of impulsive use [30].

- Inhalant-related disorders

Once again, recommendations for other DDs with inhalant-related disorders are included here. This involves using standard CBT approaches, such as assertiveness and coping skills training and alternatives to substance use. These approaches may be complemented by community reinforcement, family interventions, and an assertive approach [30].

- Behavioral addictions

There is still no research evaluating the effectiveness of specific psychological interventions for dual gambling disorder [33].

#### 3.6.1. Antisocial Personality Disorder

- Substance-related disorders

Various therapies for antisocial personality disorder (ASPD), such as CBT and CM, can help improve outcomes related to substance use patterns and abstinence maintenance, as measured by urine samples over time. However, studies on this subject are scarce and have small sample sizes [5].

A Cochrane review found that no study demonstrated significant changes in ASPD-specific behaviors, such as delinquency, aggression, and impulsivity, but several studies have found significant reductions in the use of alcohol and other drugs after treatment [9]. Some evidence supports the use of brief psychoeducational interventions and cognitive therapy [9]. It also appears that individuals benefit most from structured, behaviorally oriented therapeutic interventions [68].

Several randomized clinical trials have shown that Impulsive Lifestyle Counseling [70], a brief psychoeducational intervention, reduces substance use, improves abstinence, increases self-reported help for ASPD symptoms after three months of treatment, and reduces treatment dropout after ten months compared to usual care [9]. Furthermore, self-reported help for ASPD symptoms has been associated with improvements in both abstinence and treatment adherence [9].

Overall, the evidence suggests that psychological interventions should be the first line of treatment for individuals with dual ASPD, although the available research requires further development to determine the most effective approach [9].

- Alcohol-related disorders

There is no conclusive evidence supporting any specific psychological intervention [35].

- Opioid-related disorders

Adding CM and/or CBT to standard methadone maintenance leads to better outcomes [9]. Furthermore, adding CM to standard methadone maintenance has been associated with significantly higher therapy session attendance and improvements in social functioning [9].

- Stimulant-related disorders

CM is effective in the treatment of cocaine dependence in people with ASPD who are enrolled in a methadone maintenance program (level of evidence: 2) [13].

#### 3.6.2. Borderline Personality Disorder

- Substance-related disorders

According to Peris [68], the treatment of these DDs includes DBT [65], dual-focus schema therapy [67], and dynamic deconstructive psychotherapy [66], although there is limited evidence available. These interventions are implemented in few settings [71], likely due to their shared characteristics of a positive and consistently appreciative attitude, the need for therapists with extensive experience in both disorders, the separate provision of skills training and social therapy, and the simultaneous treatment of both disorders. Although studies show some progress in treatment over time, there is insufficient evidence to recommend one approach as preferred [72].

DBT [65] has been modified for individuals with comorbid BPD and SUDs, considering the symptoms of both BPD and substance use as attempts to regulate emotions [9]. When used to treat SUDs, DBT improves overall functioning, increases days of abstinence, and leads to negative urine samples [71]. Although the research is limited to a few studies, DBT is the preferred treatment approach because it has been demonstrated to improve both BPD symptoms and substance use [9]. In other words, DBT is effective in treating individuals with SUDs and BPD (level of evidence: 3) [13] and is particularly useful for individuals with frequent suicidal ideation [68]. In fact, in inpatient mental health services, DBT is recommended for BPD to reduce the risk of suicide, stabilize behavior, and help patients regulate their emotions [5]. Furthermore, DBT has shown similar effectiveness in group settings when administered in person and via videoconference [73].

Dual-focus schema therapy [67] may be useful for individuals who do not respond to brief interventions [68]. However, only one study has examined it, and the benefit was limited. A greater reduction in substance use was observed among those who participated in the control group, who received individual drug counseling [9]. Nevertheless, other authors consider it a promising approach that requires further investigation [5].

Dynamic deconstructive psychotherapy [66] is a modified form of psychodynamic psychotherapy initially developed for particularly complex cases of BPD, including those with comorbid SUDs [9]. Studies show that this psychotherapy significantly improves the symptoms of both BPD and SUD compared to usual treatment (community treatment). These improvements are maintained for 30 months of follow-up. It is also effective in reducing suicidal behavior [9].

Mentalization-Based Treatment (MBT) [74] is an evidence-based treatment for BPD [9]. A feasibility study found no significant differences between receiving MBT and treatment for SUD alone with respect to changes in the severity of BPD symptoms or substance use, but a trend toward a reduction in the number of suicide attempts was observed among those receiving MBT [9].

In summary, SUD treatment for individuals with BPD can be complex and progress slowly, but effective interventions exist to help reduce symptoms and improve functioning. These include strong support for DBT, dynamic deconstructive therapy, and dual-focus schema therapy for improving outcomes related to substance use, suicidal gestures and self-harm, overall and social functioning, treatment utilization, and treatment retention [5].

- Alcohol-related disorders

Three interventions have been evaluated: DBT [65], dual-focus schema therapy [67], and dynamic deconstructive therapy [66]. While all have shown promising results, they are outside of conventional clinical practice and require replication studies [69]. Thus, evidence of their effectiveness in treating this comorbidity is weak [35].

**Table 5:** Recommendations for the psychological treatment of personality disorders.

Personality Disorders		
Substance-Related Disorders	Recommended Therapies	Specific Recommendations
**Substance-related disorders**	The therapeutic alliance is the most significant predictor of outcome. Dialectical behavior therapy, dynamic deconstructive psychotherapy, and dual-focus schema therapy show promise. Treating both disorders simultaneously with psychotherapy and pharmacotherapy may be the best approach. A staged approach is recommended: first, stabilize substance use and self-harm; then, focus on interventions related to identity and self.	Schema-focused therapy is useful for treating dual personality disorders that do not respond to brief interventions.
**Alcohol-related disorders**	No evidence-based intervention was found.	
**Disorders related to sedatives, hypnotics or anxiolytics**	No evidence-based intervention was found.	
**Cannabis-related disorders**	CBT is most commonly used.	
**Stimulant-related disorders**	Effective CBT to reduce consumption.	Assistance with coping strategies can be helpful for addressing impulsive consumption.
**Inhalant-related disorders**	CBT (assertiveness and coping skills, alternatives to substance use) complemented with community reinforcement, family interventions and an assertive approach.	
**Gambling disorder**	No research is available.	
**Antisocial Personality Disorder**		
**Substance-Related Disorders**	**Recommended Therapies**	**Specific Recommendations**
**Substance-related disorders**	Psychological interventions should be the first line of treatment. CBT and contingency management may improve substance use outcomes. Cochrane review: no studies found changes in specific antisocial personality disorder behaviors, but they identified reductions in substance use. Some evidence supports the use of brief psychoeducational interventions, cognitive therapy, and structured behavioral therapy. Counseling on Impulsive Lifestyles has shown better results than usual care.	
**Alcohol-related disorders**	There is no evidence supporting a recommendation for any specific psychological intervention.	
**Opioid-related disorders**	Adding contingency management and/or CBT to standard methadone maintenance is more effective than standard maintenance alone.	
**Cocaine-related disorders**	There is no evidence supporting a recommendation for any specific psychological intervention.	Effective contingency management for dependence in individuals in a methadone maintenance program.
**Borderline Personality Disorder**		
**Substance-Related Disorders**	**Recommended Therapies**	**Specific Recommendations**
**Substance-related disorders**	Dialectical behavior therapy is effective and the preferred treatment approach. Dual-focus schema therapy, dynamic deconstructive therapy, and mentalization-based treatment show promise.	Dialectical behavior therapy is especially useful for individuals with frequent suicidal tendencies. Dual schema therapy may be helpful for individuals who do not respond to brief interventions.
**Alcohol-related disorders**	Dialectical behavior therapy, dual-focus schema therapy, and dynamic deconstructive therapy are promising.	

Note: Prepared by the authors based on [5,9,13,30,33,34,35,68,69,70,71].

## 4. Discussion

This consensus has several limitations. No quantitative measures were used to reach consensus, which may limit reliability. Most studies do not specify what the standard treatment consists of. This can lead to bias, as different studies may be comparing different treatment components grouped under the common term “standard treatment”. The same applies to “usual treatment”. Most of the available evidence on the psychological treatment of DD is level 3 or 4, and most recommendations are grade D or weak. In addition, there are several combinations of MDs and SUDs in which no evidence has been found. Similarly, there are understudied substances, MDs, populations, and barriers to treatment. Throughout the text, there may be inconsistencies in the use of the terms “integrated treatment” and “concurrent treatment”. This is because the original terms used by different authors have been retained. This limitation is common in the field of DD and reflects the need for standardization of terminology [1]. Finally, cultural, healthcare-system, and resource-related factors may influence the feasibility and generalizability of the proposed recommendations, particularly outside high-income or specialized treatment contexts.

These limitations suggest that the results of this consensus should be considered with caution. Nevertheless, this consensus, especially the tables summarizing its content, can be a useful reference tool in daily clinical practice and serve as a guide for generating new evidence. Clinicians are advised to follow the recommendations with the highest level of evidence. In cases where evidence is lacking, it is recommended to use therapies that have shown evidence for both disorders separately.

*Recommendations for the Future Research Agenda on the Psychological Treatment of Dual Disorders*

Taking into account the contents of this consensus, parts I and II, the recommendations for future research [15,24,75,76,77,78,79] that overcomes existing limitations are as follows:

1. Conduct further research into the effectiveness of integrated treatment, as well as on the timing and order of application of different treatments and/or components of psychological therapies.
2. Increase the methodological rigor of research: conduct randomized clinical trials, specify the variables and measurement instruments, use large samples with a control group, control for confounding variables (such as complementary treatments or poor delineation of patient severity), rigorously evaluate treatment adherence and the potential influence of certain aspects of the intervention on dropout rates.
3. Standardize the criteria and methods used to quantify substance use, behavioral addiction, and psychiatric symptoms.
4. Specify the substances used, behavioral addictions present, specific MDs or psychiatric symptoms exhibited by the sample and the treatment setting. Analyze the results according to all of this.
5. Conduct randomized controlled clinical trials using various comparators, such as placebo, waiting list, standard treatment, and different psychological and pharmacological treatments.
6. Conduct studies in specific populations to obtain evidence on all possible comorbidities of MDs and the different patterns of use and types of substances. In addition, consider behavioral addictions.
7. Stop excluding people with DDs from studies on MDs or SUDs and analyze the results by differentiating and comparing those of people with and without DDs.
8. Consider patient characteristics and situations, such as age, gender, geography, culture, language (different countries, ethnicities, communities, rural versus urban populations, etc.), people in the criminal justice system, people in mandatory treatment, the homeless population, people with cognitive limitations, etc. Alternatively, studies should break down the results obtained, specifying those for these populations if they were included in the participant sample.
9. Studies should specify the treatments used and their components in detail. For example, what do we mean when we talk about CBT? What are its components? What do we mean by “standard treatment”? What are its components? Use manualized therapies whenever possible and/or always specify the components included in them.
10. Study the isolated and combined effects of the different therapeutic components to verify the benefits they offer to the final outcome or lack thereof.
11. Specify, systematize, reach a consensus on, and include various outcome indicators in the studies, including those related to MDs, SUDs, quality of life, occurrence of adverse effects, adherence, and progression of disorders. Study long-term efficacy and safety. It is also important to evaluate satisfaction with the psychological treatment and its results, as well as patients’ values and preferences regarding the different therapeutic options.
12. Study the factors common to the efficacy of different psychological treatments.
13. Conduct studies that allow for the individualization of treatment, taking interpersonal variability into account, to achieve the most favorable and efficient treatment response for each patient.
14. Conduct cost-effectiveness and cost–efficacy studies.
15. Study barriers and facilitators to adopt recommendations for the psychological treatment of DDs.
16. Design and evaluate interventions in settings with limited resources and in non-specialized contexts.
17. Design and evaluate interventions to address traumatic experiences.
18. Evaluate the use of technology and e-health to facilitate more comprehensive treatment.
19. Consider patients’ perspectives, preferences, and lived experiences. For example, future updates to this consensus will include a section summarizing studies on this topic and the conclusions drawn from patient participation in DD conferences.

## 5. Conclusions

The main conclusions of the second part of this consensus are as follows:

1. Psychological treatment, particularly integrated therapy, appears to be an effective approach to treating DD in the specific comorbid disorders reviewed.
2. There is very little research on dual autism. It is recommended that interventions for SUD be adapted to the characteristics of this population.
3. More research is needed on dual social anxiety, panic, generalized anxiety, somatic symptom, and eating disorders. CBT is the most commonly used psychological treatment for these disorders.
4. For dual ADHD, a multicomponent treatment is recommended. In the first phase, PE is used, followed by individual or group CBT with peer or family support in the second phase.
5. Integrated treatments are effective for dual anxiety disorders, with CBT as the first-line treatment.
6. Trauma-focused CBT, particularly CPT and EP, is effective for dual PTSD. ACT, Stress Inoculation Training, and EMDR may also be effective.
7. Evidence is scarce for dual PDs. The most studied PDs are ASPD and BPD.
8. For dual BPD, DBT, dynamic deconstructive psychotherapy, and dual-focus schema therapy show promise.
9. For ASPD, CBT, CM, and Impulsive Lifestyle Counseling may be useful.
10. Much more empirical evidence is needed on the psychological treatment of DDs. This requires studies that overcome the methodological limitations of existing research.

## Data Availability

No new data were created.

## Abbreviations

The following abbreviations are used in this manuscript:

DD	Dual Disorder
MD	Mental Disorder
SUD	Substance Use Disorder
PTSD	Post-traumatic Stress Disorder
PD	Personality Disorder
NICE	National Institute for Health and Clinical Excellence
ADHD	Attention deficit hyperactivity disorder
PE	Psychoeducation
CBT	Cognitive Behavioral Therapy
RP	Relapse Prevention
DBT	Dialectical Behavior Therapy
MF	Mindfulness
ET	Exposure Therapy
MI	Motivational Interviewing
CPT	Cognitive Processing Therapy
PEx	Prolonged Exposure
COPE	Concurrent Treatment of Post-traumatic Stress Disorder and Substance Use Disorders Using Prolonged Exposure
EMDR	Eye Movement Desensitization and Reprocessing
ICBT	Integrated Cognitive Behavioral Therapy
ACT	Acceptance and Commitment Therapy
BPD	Borderline Personality Disorder
ASPD	Antisocial Personality Disorder
MBT	Mentalization-Based Treatment

## References

[1] Szerman N., Torrens M., Maldonado R., Balhara Y.P.S., Salom C., Maremmani I., Sher L., Didia-Attas J., Chen J., Baler R. (2022). Addictive and other mental disorders: A call for a standardized definition of dual disorders. Transl. Psychiatry.

[2] Adan A., Torrens M. (2021). Special issue: Diagnosis and management of addiction and other mental disorders (dual disorders). J. Clin. Med..

[3] Fantuzzi C., Mezzina R. (2020). Dual diagnosis: A systematic review of the organization of community health services. Int. J. Soc. Psychiatry.

[4] Scott K.D., Gorey K.M. (2025). Concurrent disorders and treatment outcomes: A meta-analysis. J. Dual Diagn..

[5] Substance Abuse and Mental Health Services Administration (2020). Substance Use Disorder Treatment for People with Co-Occurring Disorders.

[6] Torrens M., Adan A. (2023). Recent advances in dual disorders (addiction and other mental disorders). J. Clin. Med..

[7] Chetty A., Guse T., Malema M. (2023). Integrated vs non-integrated treatment outcomes in dual diagnosis disorders: A systematic review. Health SA Gesondheid.

[8] Hakobyan S., Vazirian S., Lee-Cheong S., Krausz M., Honer W.G., Schutz C.G. (2020). Concurrent disorder management guidelines: Systematic review. J. Clin. Med..

[9] Marel C., Siedlecka E., Fisher A., Gournay K., Deady M., Baker A., Kay-Lambkin F., Teesson M., Baillie A., Mills K.L. (2022). Guidelines on the Management of Co-Occurring Alcohol and Other Drug and Mental Health Conditions in Alcohol and Other Drug Treatment Settings.

[10] Asua J. (2005). Entre el consenso y la evidencia científica. Gac. Sanit..

[11] Benito A., Jiménez-Murcia S., Tirado-Muñoz J., Adan A. (2026). WADD-SEPD Consensus on psychological treatment of dual disorders I: General recommendations, most used therapies, and severe mental disorders. J. Clin. Med..

[12] American Psychiatric Association (2022). Diagnostic and Statistical Manual of Mental Disorders.

[13] Becoña E., Cortés M., Pedrero E.J., Fernández J.R., Casete L., Bermejo M.P., Secades R., Tomás V. (2008). Guía Clínica de Intervención Psicológica en Adicciones.

[14] Matthys F., Stes S., van den Brink W., Joostens P., Möbius D., Tremmery S., Sabbe B. (2014). Guideline for screening, diagnosis and treatment of ADHD in adults with substance use disorders. Int. J. Ment. Health and Addict..

[15] San L., Bernardo M., Arrojo M. (2020). Guía de Práctica Clínica: Tratamiento Farmacológico y Psicológico de los Pacientes Adultos con un Trastorno Mental Grave y un Trastorno por Uso de Sustancias.

[16] National Institute for Health and Care Excellence (NICE) (2021). Autism Spectrum Disorder in Adults: Diagnosis and Management.

[17] Crowe B.H., Salt A.T. (2015). Autism: The management and support of children and young people on the autism spectrum (NICE Clinical Guideline 170). Arch. Dis. Child.-Educ. Pract..

[18] Crunelle C.L., van den Brink W., Moggi F., Konstenius M., Franck J., Levin F.R., van de Glind G., Demetrovics Z., Coetzee C., Luderer M. (2018). International consensus statement on screening, diagnosis and treatment of substance use disorder patients with comorbid attention deficit/hyperactivity disorder. Eur. Addict. Res..

[19] Martínez-Raga J., Knecht C., Martín-Navarrete R., Szerman N., Roncero C., Casas M. (2016). Trastorno por déficit de atención con hiperactividad (TDAH). Protocolos de Intervención en Patología Dual.

[20] Young S., Woodhouse E. (2021). Assessment and treatment of substance use in adults with ADHD: A psychological approach. J. Neural Transm..

[21] Linehan M.M., Dexter-Mazza E.T., Barlow D.H. (2008). Dialectical behavior therapy for borderline personality disorder. Clinical Handbook of Psychological Disorders: A Step-by-Step Treatment Manual.

[22] National Institute for Health and Care Excellence (NICE) (2019). Attention Deficit Hyperactivity Disorder: Diagnosis and Management.

[23] van Emmerik-van Oortmerssen K., Vedel E., Koeter M.W., de Bruijn K., Dekker J.J., van den Brink W., Schoecers R.A. (2013). Investigating the efficacy of integrated cognitive behavioral therapy for adult treatment-seeking substance use disorder patients with comorbid ADHD: Study protocol of a randomized controlled trial. BMC Psychiatry.

[24] Preuss U.W., Gouzoulis-Mayfrank E., Havemann-Reinecke U., Schäfer I., Beutel M., Hoch E., Mann K.F. (2018). Psychiatric comorbidity in alcohol use disorders: Results from the German S3 guidelines. Eur. Arch. Psychiatry Clin. Neurosci..

[25] Sáiz Martínez P.A., Jiménez Treviño L., Díaz Mesa E.M., García-Portilla González M.P., Marina González P., Al-Halabí S., Szerman N., Bobes García J., Ruiz P. (2014). Patología dual en trastornos de ansiedad: Recomendaciones en el tratamiento farmacológico [Dual diagnosis in anxiety disorders: Pharmacologic treatment recommendations]. Adicciones.

[26] Saiz P., Jiménez-Treviño L., Menéndez I., García-Portilla M.P., Bobes J., Flórez G., Gómez-Trigo J., Szerman N., Roncero C., Casas M. (2016). Patología dual en ansiedad. Protocolos de Intervención en Patología Dual.

[27] Queensland Health (2010). Dual Diagnosis Clinical Guidelines: Co-Occurring Mental Health and Alcohol and Other Drug Problems.

[28] Antai-Otong D., Theis K., Patrick D.D. (2016). Dual diagnosis: Coexisting substance use disorders and psychiatric disorders. Nurs. Clin. N. Am..

[29] Murthy P., Chand P. (2012). Treatment of dual diagnosis disorders. Curr. Opin. Psychiatry.

[30] Gordon A. (2008). Comorbidity of Mental Disorders and Substance Use: A Brief Guide for the Primary Care Clinician.

[31] Miller W.R., Rollnick S. (1999). La Entrevista Motivacional: Preparar Para el Cambio de Conductas Adictivas.

[32] Mocanu V., Wood E. (2022). Alcohol use disorder with comorbid anxiety disorder: A case report and focused literature review. Addict. Sci. Clin. Pract..

[33] Fernández J.J., Orengo T., Díaz S. (2017). Comorbilidad Psiquiátrica en Adicciones. Trastorno por Uso de Opioides y Otros Trastornos Mentales. Guías Clínicas Basadas en la Evidencia. Trastornos por Uso de Sustancias y Otros Trastornos Mentales.

[34] Guardia J., Flórez G. (2017). Comorbilidad Psiquiátrica en Adicciones. Trastorno por Uso de Ansiolíticos e Hipnóticos y Otros Trastornos Mentales. Guías Clínicas Basadas en la Evidencia. Trastornos por Uso de Sustancias y Otros Trastornos Mentales.

[35] Flórez G., Balcells M., Uzal C. (2017). Comorbilidad Psiquiátrica en Adicciones. Trastorno por Uso de Alcohol y Trastorno Mental Comórbido. Guías Clínicas Basadas en la Evidencia. Trastornos por Uso de Sustancias y Otros Trastornos Mentales.

[36] Simpson T.L., Lehavot K., Petrakis I.L. (2017). No wrong doors: Findings from a critical review of behavioral randomized clinical trials for individuals with co-occurring alcohol/drug problems and posttraumatic stress disorder. Alcohol. Clin. Exp. Res..

[37] Resick P.A., Monson C.M., Chard K.M. (2016). Cognitive Processing Therapy for PTSD: A Comprehensive Manual.

[38] Foa E.B., Hembree E.A., Rothbaum B.O. (2007). Prolonged Exposure Therapy for PTSD: Emotional Processing of Traumatic Experiences Therapist Guide.

[39] Somohano V.C., Cameron D., Lewis M., Denneson L.M., Lovejoy T.I., O’Neil M.E. (2024). Characterizing and comparing evidence-based psychotherapy utilization among veterans with co-occurring PTSD and substance use disorder. Subst. Use Misuse.

[40] Back S.E., Foa E.B., Killeen T.K., Mills K.L., Teesson M., Cotton B.D., Carroll K.M., Brady K.T. (2014). Concurrent Treatment of PTSD and Substance Use Disorders Using Prolonged Exposure (COPE).

[41] Foa E.B., Yusko D.A., McLean C.P., Suvak M.K., Bux D.A., Oslin D., O’Brien C.P., Imms P., Riggs D.S., Volpicelli J. (2013). Concurrent naltrexone and prolonged exposure therapy for patients with comorbid alcohol dependence and PTSD: A randomized clinical trial. JAMA.

[42] Najavits L.M., Weiss R.D., Shaw S.R., Muenz L.R. (1998). “Seeking safety”: Outcome of a new cognitive-behavioral psychotherapy for women with posttraumatic stress disorder and substance dependence. J. Trauma. Stress.

[43] Bernardy N.C., Hamblen J.L., Friedman M.J., Kivlahan D.R. (2011). Co-occurring posttraumatic stress disorder and substance use disorder: Recommendations for management and implementation in the Department of Veterans Affairs. J. Dual Diagn..

[44] Shapiro F. (1989). Efficacy of the Eye Movement Desensitization procedure in the treatment of traumatic memories. J. Trauma. Stress.

[45] Triffleman E.G., Carroll K., Kellogg S. (1999). Substance dependence posttraumatic stress disorder therapy: An integrated cognitive-behavioral approach. J. Subst. Abuse Treat..

[46] Back S.E., Dansky B.S., Carroll K.M., Foa E.B., Brady K.T. (2001). Exposure therapy in the treatment of PTSD among cocaine-dependent individuals: Description of procedures. J. Subst. Abuse Treat..

[47] Brady K.T., Dansky B.S., Back S.E., Foa E.B., Carroll K.M. (2001). Exposure therapy in the treatment of PTSD among cocaine-dependent individuals: Preliminary findings. J. Subst. Abuse Treat..

[48] Saunders E.C., McGovern M.P., Capone C., Hamblen J., Vujanovic A.A., Back S.E. (2019). Integrated cognitive-behavioral therapy (ICBT) for co-occurring PTSD and substance use disorders: Overview and new applications. Posttraumatic Stress and Substance Use Disorders.

[49] Garland E.L., Hanley A.W., Riquino M.R., Reese S.E., Baker A.K., Salas K., Howard M.O. (2019). Mindfulness-oriented recovery enhancement reduces opioid misuse risk via analgesic and positive psychological mechanisms: A randomized controlled trial. J. Consult. Clin. Psychol..

[50] Eaton E., Capone C., Reese S., Shea M.T., Serpa J.G., Germer C. (2025). Mindful self-compassion for veterans with morally injurious experiences and co-occurring posttraumatic stress disorder and substance use disorder: A feasibility study. J. Dual Diagn..

[51] McGovern M.P., Lambert-Harris C., Acquilano S., Xie H., Alterman A.I., Weiss R.D. (2009). A cognitive behavioral therapy for co-occurring substance use and posttraumatic stress disorders. Addict. Behav..

[52] Najavits L.M. (2002). Seeking Safety: A Treatment Manual for PTSD and Substance Abuse.

[53] Somohano V.C., Bowen S. (2022). Trauma-integrated mindfulness-based relapse prevention for women with comorbid post-traumatic stress disorder and substance use disorder: A cluster randomized controlled feasibility and acceptability trial. J. Integr. Complement. Med..

[54] Danielson C.K., McCart M.R., de Arellano M.A., Macdonald A., Doherty L.S., Resnick H.S. (2010). Risk reduction for substance use and trauma-related psychopathology in adolescent sexual assault victims: Findings from an open trial. Child Maltreat..

[55] Hien D.A., Fitzpatrick S., Saavedra L.M., Ebrahimi C.T., Norman S.B., Tripp J., Ruglass L.M., Lopez-Castro T., Killeen T.K., Back S.E. (2021). What’s in a name? A data-driven method to identify optimal psychotherapy classifications to advance treatment research on co-occurring PTSD and substance use disorders. Eur. J. Psychotraumatol..

[56] Hayes S.C., Strosahl K.D., Wilson K.G. (2012). Acceptance and Commitment Therapy: The Process and Practice of Mindful Change.

[57] Taylor M., Petrakis I., Ralevski E. (2017). Treatment of alcohol use disorder and co-occurring PTSD. Am. J. Drug Alcohol Abuse.

[58] Courbasson C.M., Nishikawa Y., Shapira L.B. (2010). Mindfulness-action based cognitive behavioral therapy for concurrent binge eating disorder and substance use disorders. Eat. Disord..

[59] Courbasson C.M., Nishikawa Y., Dixon L. (2012). Outcome of dialectical behaviour therapy for concurrent eating and substance use disorders. Clin. Psychol. Psychother..

[60] Sugarman D.E., Meyer L.E., Reilly M.E., King B.R., Dechant E., Weigel T., Tarbox P., Greenfield S.F. (2020). The women’s recovery group for individuals with co-occurring substance use and eating disorders: Feasibility and satisfaction in a residential eating disorders program. Alcohol. Treat. Q..

[61] Humphreys E., Ladner T., van Draanen J. (2023). A qualitative assessment of the treatment needs of women with concurrent eating and substance use disorders in a residential setting. J. Dual Diagn..

[62] Becoña E., Cortés M., Arias F., Barreiro C., Berdullas J., Iraurgui J., Llorente J.M., López A., Madoz A., Martínez J.M. (2011). Manual de Adicciones Para Psicólogos Especialistas en Psicología Clínica en Formación.

[63] Martínez-González J.M., Trujillo H.M. (2003). Tratamiento del Drogodependiente con Trastornos de la Personalidad.

[64] Martínez J.M. (2010). Psychological intervention in patients with comorbid cocaine and alcohol and personality disorders. Trastor. Adict..

[65] Linehan M.M., Schmidt H., Dimeff L.A., Craft J.C., Kanter J., Comtois K.A. (1999). Dialectical behavior therapy for patients with borderline personality disorder and drug dependence. Am. J. Addict..

[66] Gregory R.J., Remmen A.L. (2008). A manual-based psychodynamic therapy for treatment-resistant borderline personality disorder. Psychother. Theory Res. Pract..

[67] Young J., Klosko J., Weishaar M. (2003). Schema Therapy: A Practitioner’s Guide.

[68] Peris L., Szerman N., Roncero C., Casas M. (2016). Patología dual en trastornos de personalidad. Protocolos de Intervención en Patología Dual.

[69] Newton-Howes G., Foulds J. (2018). Personality disorder and treatment outcome in alcohol use disorder. Curr. Opin. Psychiatry.

[70] Thylstrup B., Hesse M. (2011). The impulsive lifestyle counseling program for antisocial behavior in outpatient substance abuse treatment. Int. J. Offender Ther. Comp. Criminol..

[71] Kienast T., Stoffers J., Bermpohl F., Lieb K. (2014). Borderline personality disorder and comorbid addiction: Epidemiology and treatment. Dtsch. Ärzteblatt Int..

[72] Pennay A., Cameron J., Reichert T., Strickland H., Lee N.K., Hall K., Lubman D.I. (2011). A systematic review of interventions for co-occurring substance use disorder and borderline personality disorder. J. Subst. Abuse Treat..

[73] Bean C.A.L., Aurora P., Maddox C.J., Mekota R., Updegraff A. (2022). A comparison of telehealth versus in-person group therapy: Results from a DBT-based dual diagnosis IOP. J. Clin. Psychol..

[74] Bateman A., Fonagy P. (2006). Mentalization-Based Treatment for Borderline Personality Disorder: A Practical Guide.

[75] Dass-Brailsford P., Myrick A.C. (2010). Psychological trauma and substance abuse: The need for an integrated approach. Trauma. Violence Abuse.

[76] Murthy P., Mahadevan J., Chand P.K. (2019). Treatment of substance use disorders with co-occurring severe mental health disorders. Curr. Opin. Psychiatry.

[77] Secades-Álvarez A., Fernández-Rodríguez C. (2017). Review of the efficacy of treatments for bipolar disorder and substance abuse. Rev. Psiquiatr. Salud Ment..

[78] Subodh B.N., Sharma N., Shah R. (2018). Psychosocial interventions in patients with dual diagnosis. Indian J. Psychiatry.

[79] Szerman N., Peris L. (2018). Precision psychiatry and dual disorders. J. Dual Diagn..

